# Improvement of PHBV Scaffolds with Bioglass for Cartilage Tissue Engineering

**DOI:** 10.1371/journal.pone.0071563

**Published:** 2013-08-09

**Authors:** Jun Wu, Ke Xue, Haiyan Li, Junying Sun, Kai Liu

**Affiliations:** 1 Department of Orthopedics, the First Affiliated Hospital of Soochow University, Jiangsu Suzhou, China; 2 Department of Orthopedics, the First People’s Hospital of Changzhou, Jiangsu Changzhou, China; 3 Department of Plastic and Reconstructive Surgery, Shanghai 9th People’s Hospital, Shanghai Jiao Tong University School of Medicine, Shanghai, China; 4 Med-X Research Institute, School of Biomedical Engineering, Shanghai Jiao Tong University, Shanghai, China; Instituto de Engenharia Biomédica, University of Porto, Portugal

## Abstract

Polymer scaffold systems consisting of poly(hydroxybutyrate-co-hydroxyvalerate) (PHBV) have proven to be possible matrices for the three-dimensional growth of chondrocyte cultures. However, the engineered cartilage grown on these PHBV scaffolds is currently unsatisfactory for clinical applications due to PHBV’s poor hydrophilicity, resulting in inadequate thickness and poor biomechanical properties of the engineered cartilage. It has been reported that the incorporation of Bioglass (BG) into PHBV can improve the hydrophilicity of the composites. In this study, we compared the effects of PHBV scaffolds and PHBV/BG composite scaffolds on the properties of engineered cartilage *in vivo*. Rabbit articular chondrocytes were seeded into PHBV scaffolds and PHBV/BG scaffolds. Short-term in vitro culture followed by long-term *in vivo* transplantation was performed to evaluate the difference in cartilage regeneration between the cartilage layers grown on PHBV and PHBV/BG scaffolds. The results show that the incorporation of BG into PHBV efficiently improved both the hydrophilicity of the composites and the percentage of adhered cells and promoted cell migration into the inner part the constructs. With prolonged incubation time *in vivo*, the chondrocyte-scaffold constructs in the PHBV/BG group formed thicker cartilage-like tissue with better biomechanical properties and a higher cartilage matrix content than the constructs in the PHBV/BG group. These results indicate that PHBV/BG scaffolds can be used to prepare better engineered cartilage than pure PHBV.

## Introduction

The production of cartilage using tissue engineering provides new approaches for the meeting clinical needs associated with articular repair. Growing isolated chondrocytes on polymeric scaffolds to construct three-dimensional articular cartilage tissue for implantation is a primary approach in tissue engineering involving the regeneration of tissue [1]–[4]. A variety of biodegradable polymers have been explored for the repair of cartilage defects either alone or as scaffolds for cartilage tissue engineering. These polymers include collagen gels and sponges [3], hyaluronic acid matrices [5] and poly(a-hydroxyesters) such as poly(glycolic acid) (PGA), poly(lactic acid) (PLA) and their copolymers [6]–[8]. Novel polyhydroxyalkanoates (PHAs) are biodegradable, biocompatible and thermoplastic polyesters produced by various microorganisms and by genetically modified plants [9]. These polymers are ideal for use as biomedical materials due to their unique and interesting physicochemical features in addition to their mechanical properties, which are similar to those of polypropylene and polylactic-co-glycolic acids [10]. The most extensively studied PHA is poly(3-hydroxybutyrate) (PHB), which can be produced in high yield by fermentation with a variety of bacterial strains. Its copolymers with varying ratios of hydroxyvalerate (PHBV) are the most widely used materials because they are less crystalline, more flexible and more readily processable than PHB itself.

Many studies have used PHB and PHBV as biomaterials for cartilage tissue engineering [11]–[13]. Deng et al. investigated the proliferation of rabbit articular chondrocytes on polymer scaffold systems consisting poly(hydroxybutyrate-co-hydroxyhexanoate) (PHBHHx)/polyhydroxybutyrate (PHB), and the results showed that the chondrocytes proliferated on the scaffolds and preserved their phenotype for up to 28 days [12]. Kose et al. used macroporous PHBV matrices for cartilage tissue engineering to repair full-thickness cartilage defects (4.5 mm side length and 4 mm depth) in rabbits, and the *in vivo* results showed that chondrocytes seeded into PHBV matrices exhibited early cartilage formation resembling normal articular cartilage and that there was only a minimal foreign body reaction at 8 and 20 weeks [14]. Liu et al. used PHBV and predifferentiated human adipose-derived stem cells (hASCs) for cartilage tissue engineering and found that chondrogenic predifferentiated hASCs have the ability to maintain a chondrogenic phenotype in PHBV and that cell/PHBV constructs can produce neocartilage in a heterotopic site [15]. PHBV is a type of hydrophobic polyester. In our previous study, we used PHBV as a scaffold for cartilage tissue engineering and showed that PHBV scaffolds have the potential to be used as chondrocyte carriers for cartilage engineering [16]. However, we found that PHBV has poor hydrophilicity, which resulted in a low percentage of adhered cells.

Bioglass® (BG) is a bioactive inorganic material composed of SiO_2_, Na_2_O, CaO and P_2_O_5_ in specific proportions. 45S5 is the original composition of Bioglass® [17]. It has been reported that the incorporation of 45S5 BG into PHBV can improve the hydrophilicity of the composites, and the level of enhancement is dependent on the 45S5 BG content in the composites [17]. However, to date, few studies have tested whether this method could be used in cartilage tissue engineering.

Therefore, in this study, BG 45S5 was incorporated into PHBV, and PHBV and PHBV/BG composite scaffolds were prepared. Chondrocytes were seeded into the scaffolds and cultured *in vitro* for 3 weeks, followed by long-term *in vivo* implantation to observe the growth and proliferation of the chondrocytes and engineered cartilage tissue on different scaffolds. The cell migration in different scaffolds, extracellular matrix production, and structure, size and biomechanical properties of the neocartilage were analyzed to evaluate the effects of the incorporation of BG into PHBV on the structure and function of the regenerated cartilage.

## Materials and Methods

All animal experimental procedures in this study were approved by the Ethics Committee of the Soochow University School of Medicine.

### Preparation of PHBV and PHBV/BG Scaffolds

Poly(3-hydroxybutyrate-co-3-hydroxyvalerate) (PHBV, Mw = 300000) containing 3 mol% 3-hydroxyvalerate units was obtained from Tianan Biologic Material Co. Ltd. (Ninbo, China). PHBV and PHBV/BG composite scaffolds containing 20% BG (w/w) were prepared using a solvent casting-particulate leaching method as described previously [18]. Briefly, 1 g of PHBV powder was dissolved in 10 ml of chloroform to give a concentration of 10% (w/v). For the PHBV/BG composite scaffolds, 0.25 g of BG powder was added to the solution with continuous stirring for 2 h to uniformly disperse the BG powder. Sodium chloride (NaCl) particles were then added to the mixture as porogens. The mixture was cast in a Teflon mold with a diameter of 60 mm and a height of 3 mm. After being air-dried in a fume hood for 24 h to allow the solvent to evaporate, the samples were vacuum-dried at 60°C for 48 h to remove any remaining solvent. The dried samples were then immersed in deionized water to leach out the porogens. Lastly, the samples were vacuum-dried again to obtain porous scaffolds. The as-prepared samples are referred to as PHBV and PHBV/BG scaffolds. Scaffolds cut into the shape of rectangular prisms with the same size (4 mm side length, 3 mm thick) were used in the study. To determine the water contact angle, PHBV and PHBV/20% BG films were prepared using the same method without the addition of the porogen or the particulate leaching process. The AgNO3 titration method was used to determine if the NaCl had been completely leached out of the scaffolds [18].

### Characterization of the PHBV and PHBV/BG Scaffolds

The PHBV and PHBV/BG scaffolds were first evaluated by optical microscopy and were then examined with a scanning electron microscope (SEM; EPMA-8705QH2, Shimadzu, Japan) after being coated with gold. The SEM examination was performed at an accelerating voltage of 20 kV. To expose the internal architecture, a sample was cut carefully with a razor blade after being vacuum dried. The SEM images of the scaffolds were analyzed using image analysis software to obtain the pore size data. Over forty results were averaged to obtain the mean value and standard deviation of the pore size. The porosity of the porous scaffolds was determined by measuring the dimensions and mass of the scaffolds as described by Hou et al. [19]. For compressive strength testing, PHBV and PHBV/BG scaffolds with dimensions of 6 mm in diameter and 3 mm thick were prepared, and the compressive strength was determined with an AG-1 Shimadzu mechanical tester (Shimadzu Co., Japan). The crosshead speed was 0.5 mm/min.

The water absorptivity of the scaffolds was determined using the methods previously reported [20]. The completely dried scaffolds were weighed (Wdry) and then placed in deionized water to allow water absorption equilibration at room temperature for 4 h. Then, the hydrated scaffolds were removed from the water, and the free surface water was removed using filter paper. Then the hydrated scaffolds were weighed (Wwet), and the water absorption was calculated using the following formula: water absorption ratio (%) = (Wwet – Wdry)/Wdry×100%.

### Hydrophilicity Determination

The hydrophilicity of the composites was evaluated by measuring the water contact angles of the nonporous composite cuboids. The water contact angles were determined using the sessile drop method at 25°C using a contact angle goniometer (model SZ10-JC2000A. Shanghai, China). The contact angles were measured at five different locations for each specimen. For each location, a 0.5 µL deionized water drop was deposited onto the surface. The degree of reproducibility for the different specimens was within ±4.0°. Three specimens were tested for each sample.

### Cell Isolation and Construction of Engineered Cartilage

The articular cartilage was removed from the knee and hip joints of young (3–5 days old) New Zealand white rabbits and cut into small pieces as previously described [21]. Chondrocytes were released from the cartilage slices by digestion with (0.2% w/v) collagenase II. The isolated cells were then cultured in DMEM supplemented with 10% fetal calf serum (FCS), 100 U/ml penicillin, and 100 ug/ml streptomycin. The cells were incubated at 37°C in a 5% CO_2_ incubator, and the medium was changed every 3 days. After the cells had been subcultured twice, 30 µl (5×10^7 ^cell/ml) of the chondrocyte suspension at passage 2 was dropped onto each scaffold. Inoculation was performed in 6-well polystyrene culture plates. The scaffolds were placed in the center of the wells, and 5 ml of cell suspension was added to each well to allow full attachment of the cells to the scaffolds. The culture medium was changed every 3 days, and the constructs were harvested at 3 weeks and implanted subcutaneously into nude mice. The specimens were harvested at 4, 8, and 12 weeks post-implantation.

### Fluorescence Analysis of Cell Migration

A chondrocyte line labeled with green fluorescent protein (GFP) was used to examine cell migration on different scaffolds at different time points (4 hours and 12 hours after cell seeding on the scaffolds). The constructs at the different culture time points were fixed in formaldehyde at room temperature for 2 h, embedded in SAKURA Tissue-Tek® O.C.T. Compound (USA), and sectioned at 5 mm on a freezing microtome (Shandon Cryotome E, Pittsburgh, USA). Then, the sections were observed under a fluorescence microscope.

### Cell Adhesion and Proliferation

The PHBV and PHBV/BG composite substrates were soaked in 75% ethanol for 48 h and sterilized with ultraviolet radiation overnight, followed by washing with sterile phosphate-buffered saline (PBS, pH 7.4). Then, the chondrocytes were seeded onto the substrates in a 48-well plate at a density of 70 cells/mm^2^. The chondrocytes were maintained in a CO_2_ incubator for 3 h before 1 ml of fresh medium was added to each well. The number of living cells was measured using an MTT-based colorimetric assay to determine the percentage of adhered cells as previously described [22].

The number of cells on different scaffolds was measured based on the DNA content of each sample after being cultured *in vitro* for different periods of time [23]. The *MTT* assay *was used* to *assess* the effects of different scaffolds on cell proliferation. A PHBV/BG scaffold was added to Dulbecco’s modified Eagle’s medium (DMEM) and incubated in an incubator at 37°C for 24 h. The DMEM with PHBV scaffold treatment was used as the control. Chondrocytes were cultured in the above medium for 2 days, 4 days, and 6 days and then analyzed with the MTT assay.

### The Evaluation of in vivo Engineered Tissue

#### Gross observation of in vivo engineered tissue

The constructs harvested at 4 weeks, 8 weeks, and 12 weeks after implantation were photographed to determine the side length (S), volume (V) and thickness (T). The volume was determined using a volumenometer, and the side length and thickness were measured using a vernier caliper at each time point.

#### Quantitative analysis of in vivo cartilage formation

After 4, 8, and 12 weeks of *in vivo* culture, the wet weight, glycosaminoglycan (GAG) content [24], [25], and total collagen content [26] were determined according to previously described methods.

#### Biomechanical analysis

A biomechanical analyzer (Instron, Canton, MA, USA) was used for biomechanical testing. As previously described [27], a constant compressive strain rate of 1 mm/min was applied until a maximal force of 100 N was achieved, and a force-displacement curve was obtained. The compressive modulus of the tested tissue was calculated based on the force-displacement curve.

#### Histology evaluation

After implantation for 4, 8 or 12 weeks, representative *in vivo*-formed cartilaginous tissue on PHBV and PHBV/BG scaffolds was fixed in neutral buffered formalin, embedded in paraffin, and sectioned (10-µm thickness). The cross-sections were stained with hematoxylin and eosin. Ten-micrometer cryosections were used for immunostaining with a type II collagen antibody. The samples were immersed in PBS containing 1% goat serum at 4°C overnight to block non-specific binding sites. Subsequently, the sections were incubated in PBS containing 1% BSA and an anti-type II collagen antibody (1∶100 working dilution) at 25°C for 4 h. After being washed with PBS three times, the samples were incubated in PBS containing 3% BSA. Lastly, the samples were incubated in PBS containing 1% BSA and a horseradish peroxidase (HRP)-conjugated anti-rabbit IgG antibody (1∶150 working dilution) at 25°C for 4 h, followed by color development with diaminobenzidine tetrahydrochloride (DAB, Santa Cruz).

### Statistical Analysis

All data were expressed as the means ± standard deviation (SD) for n  = 6. The differences between the PHBV and PHBV/BG scaffolds with respect to the volume, porosity, compressive strength, water absorptivity, water contact angles, and cell adhesion were analyzed by Student’s t-test. The differences in the DNA content, cell proliferation, thickness, side length, volume, wet weight, compressive moduli, collagen content, GAG content, and comp. modulus of the in vivo specimens among various time points between the two groups were assessed by two-way ANOVA. A p-value less than 0.05 was considered to be statistically significant.

## Results

### Characterization of the Scaffolds

**Figure 1:**
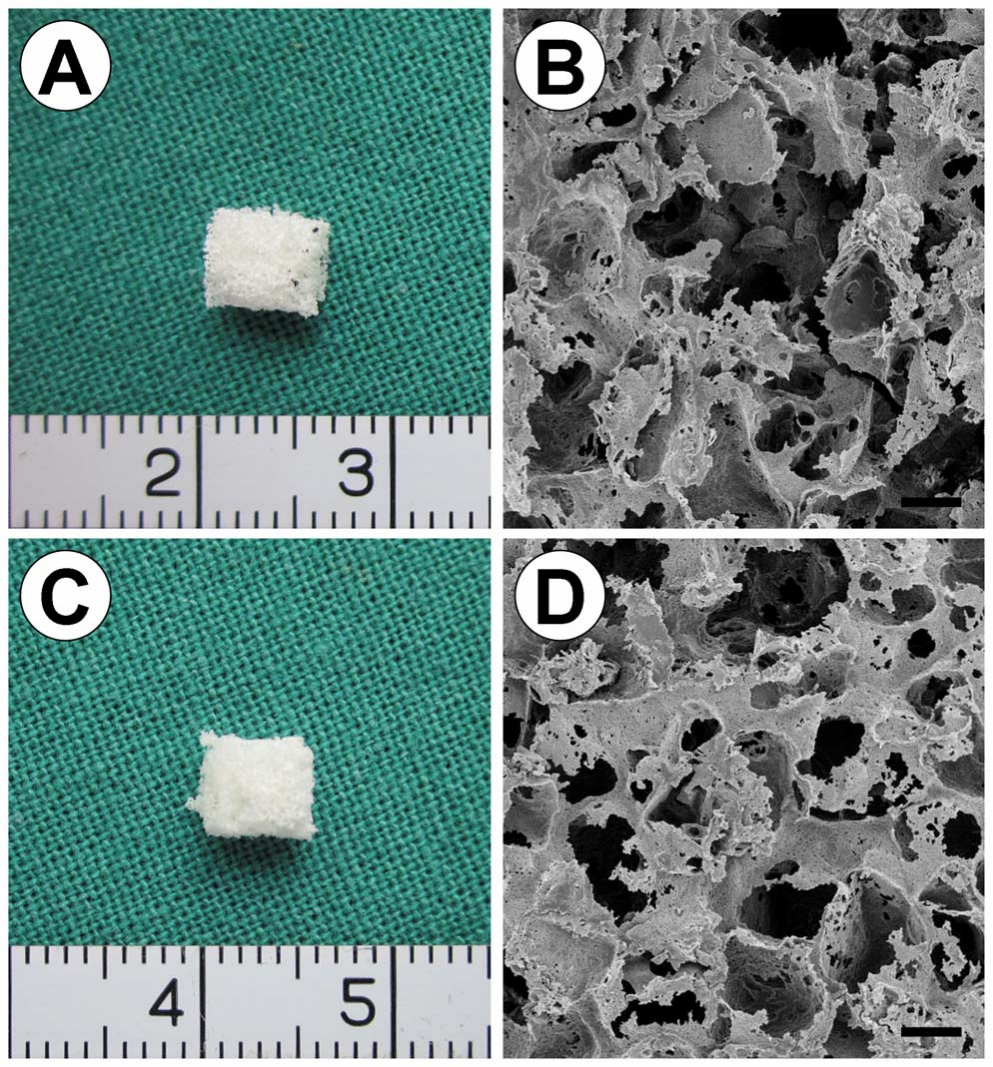
Optical and SEM micrographs of the PHBV and PHBV/BG scaffolds. The PHBV and PHBV/BG scaffolds prepared using the compression molding, thermal processing and salt particulate leaching method had a regular shape, and some pores on the surface of the scaffolds can be observed. The SEM images show that the PHBV and PHBV/BG scaffolds exhibited a macroporous structure with interconnected open pores, and the pore size varied from 30 µm to 300 µm. Scale bar  = 100 µm.

**Figure 2:**
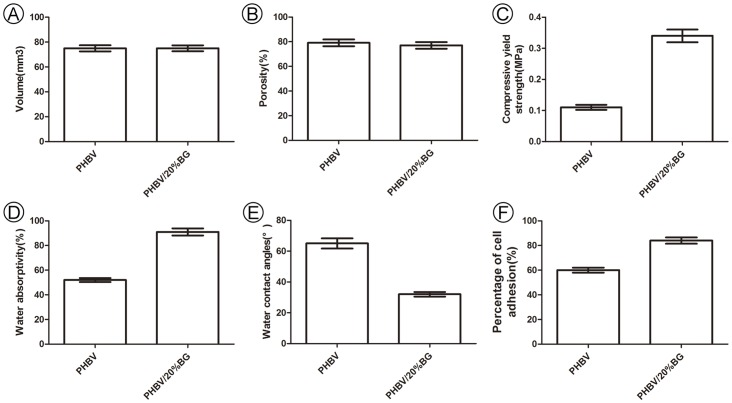
Characterization of the scaffolds. As shown in Fig. 2, both the PHBV and PHBV/BG scaffolds were fabricated in the shape of rectangular prisms with the same volume and the same porosity (p>0.05). However, there were significant differences between PHBV and PHBV/BG in terms of the compressive strength, water absorptivity, water contact angle and cell adhesion.

As shown in Fig. 1, both the PHBV (Fig. 1A) and PHBV/BG (Fig. 1C) scaffolds were cut into the shape of rectangular prisms with the same size (4 mm side length, 3 mm thick) and the same volume (p>0.05). SEM analysis showed that the PHBV and PHBV/BG scaffolds had macroporous structures with interconnected open pores, and the pore size varied from 30 µm to 300 µm (Fig. 1B, 1D). The scaffolds in both groups also had similar porosities (p>0.05) (Fig. 2B). These results indicate that the addition of BG to PHBV did not change the micro or macro structure of the scaffold. However, the compressive strength and the water absorptivity of the scaffolds increased from 0.11±0.02 MPa to 0.34±0.05 MPa from 52±3% to 91±5%, respectively, with increasing BG content from 0 to 20 wt% (p < 0.05)(Fig. 2C, 2D).

### Hydrophilicity of Different Scaffolds

Fig. 2E shows that the water contact angles of the PHBV/BG composites (32±1.5°) were significantly lower than those of PHBV (65±2°) (pure PHBV) (p<0.01), suggesting that there was an obvious improvement in surface hydrophilicity with the addition of BG to PHBV.

### Cell Migration in Different Scaffolds

**Figure 3:**
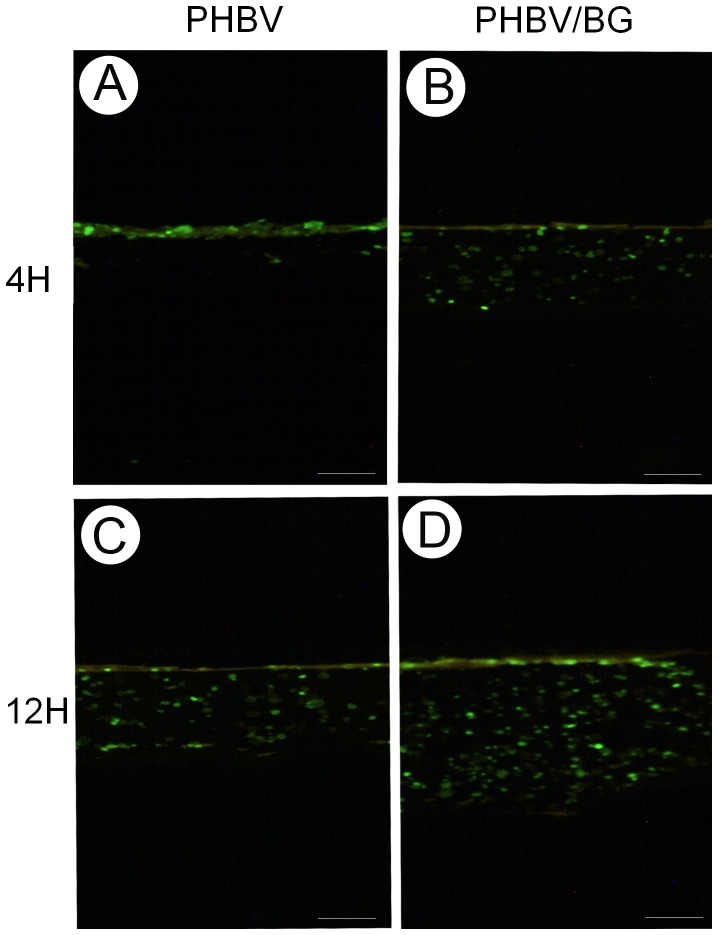
Fluorescence analysis of cell migration. GFP labeling showed that the cells could easily migrate toward the inner region of the scaffolds in in vitro culture in the PHBV/BG group. In the PHBV group, the cells primarily assembled on the surface of the scaffolds, even after 12 h of culture. Scale bar  = 100 µm.

Cell migration in the scaffolds was continuously monitored over 12 h by fluorescence microscopy (Fig. 3). In the PHBV/BG group, cells exhibited superior migration toward the inner region of the scaffolds with increasing in vitro culture time. However, in the PHBV group, the cells assembled primarily on the surface of the scaffolds within 12 h. Evidently, these results were closely related to the water absorptivity of the scaffolds.

### Cell Adhesion and Proliferation

**Figure 4:**
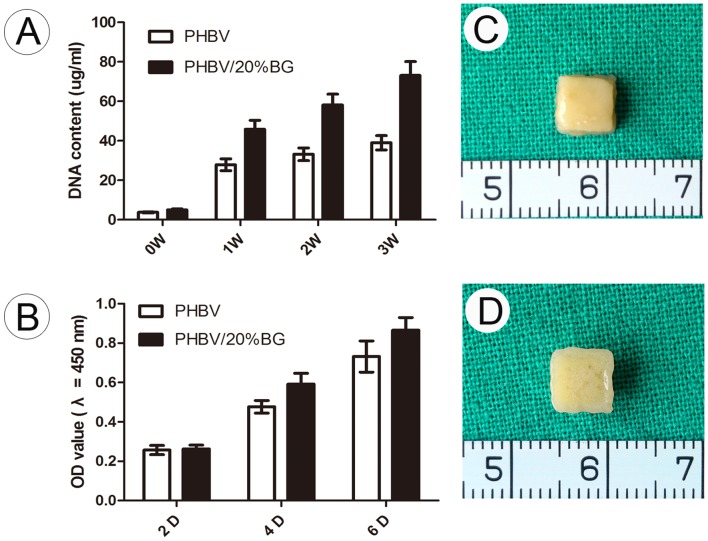
Gross view of tissue engineered cartilage and cell proliferation for each group. The DNA content of the cells retrieved from the different scaffolds at different time points during in vitro culture was determined. The DNA content of the PHBV/BG group was significantly higher than that of the PHBV group. The results of the MTT assay showed that the incorporation of BG enhanced chondrocyte proliferation. The chondrocyte-scaffold constructs were harvested after 3 weeks of culture *in vitro*, and it was observed that the chondrocyte-scaffold constructs formed cartilage-like tissue.

The percentage of adhered cells increased from 60±5% to 84±7% with the addition of BG (from 0 to 20 wt%) (Fig. 2F). In addition, the DNA content of the cells cultured *in vitro* on different materials for 3 weeks before subcutaneous transplantation was measured. There were significant differences in the DNA content of cells cultured on PHBV and those cultured on the PHBV/BG composite (Fig. 4A). The MTT results indicate that the PHBV/BG group had better cellular proliferation than the PHBV group (Fig. 4B).

### The Evaluation of *in vivo* Engineered Tissue Produced with Different Scaffolds

#### Gross evaluation of in vivo engineered constructs

**Figure 5:**
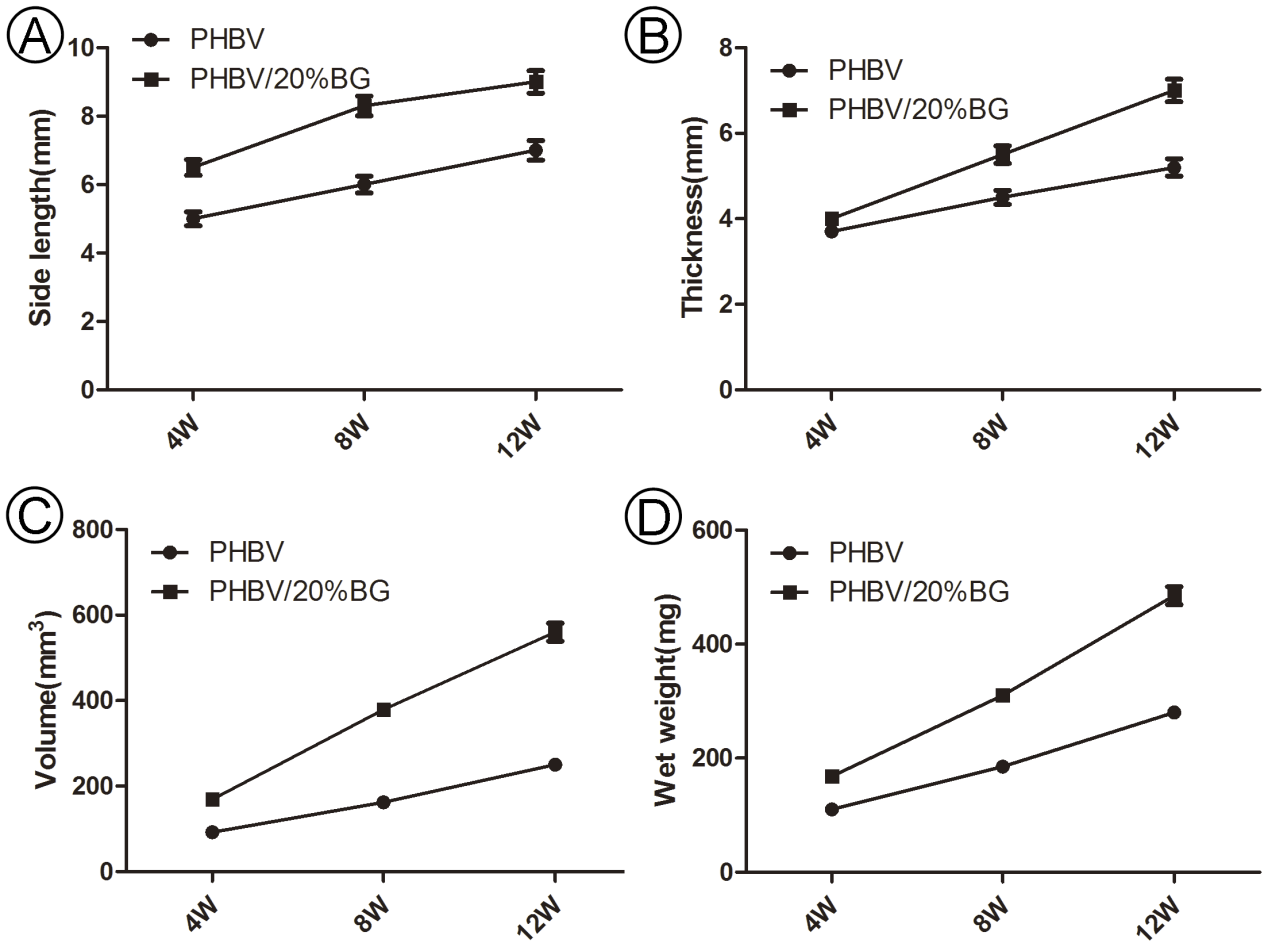
Gross evaluation of *in vivo* engineered constructs. There were significant differences between the two groups in terms of the thickness, side length, volume and wet weight from 4 to 12 weeks *in vivo* (p<0.05). These results indicate that the incorporation of BG into PHBV can promote the adhesion and proliferation of chondrocytes on scaffolds.

The variations of the *in vivo* engineered constructs in gross view and size were recorded to roughly evaluate the effects of the different scaffolds on 3D tissue formation. After being implanted *in vivo*, the constructs in the two groups basically maintained their original size and presented a cartilage-like appearance. Quantitative analysis indicated that there was a significant difference between the regenerated cartilage samples of two groups in terms of thickness, side length, volume and wet weight (Fig. 5) from 4 to 12 weeks (p<0.05).

#### Collagen content, GAG content and comp. modulus

**Figure 6:**
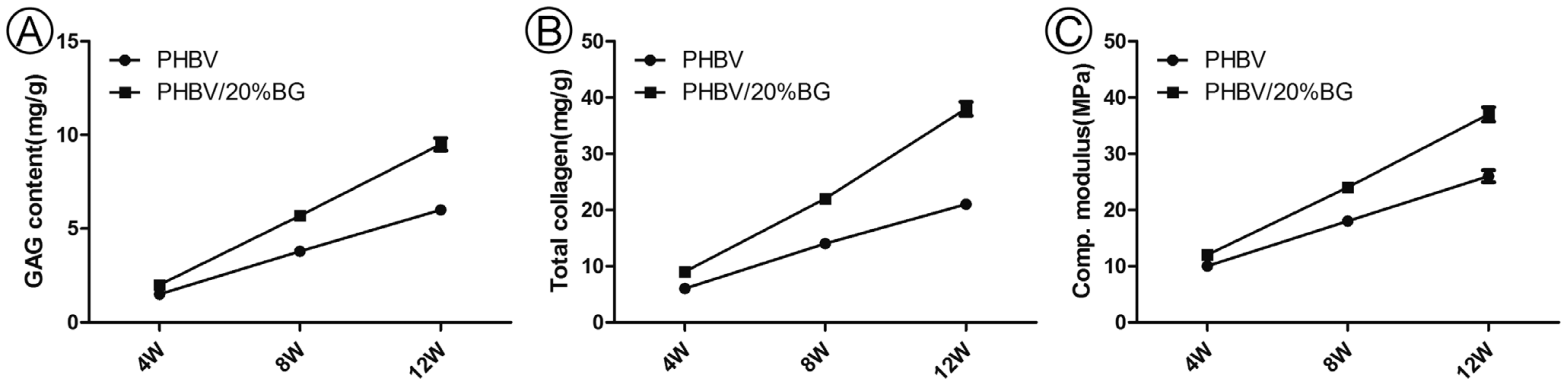
The collagen content, GAG content and comp. modulus. The collagen and GAG contents and the comp. modulus were significantly higher in the PHBV/BG group than in the PHBV group at different time points (p<0.05).

Quantitative analysis further demonstrated that the ECM (extracellular matrix) content in the PHBV/BG group was significantly higher than that in the PHBV group at different time points (p<0.05) (Fig. 6A, 6B).

The mechanical properties of the cartilage produced are important, and the collagen and GAG contents at least partly reflect these properties. Therefore, these three parameters were evaluated in this study. As we expected, the collagen and GAG contents and the comp. modulus were also significantly higher in the PHBV/BG group than in the PHBV group at different time points (p<0.05) (Fig. 6).

#### Histology and immunohistochemistry

**Figure 7:**
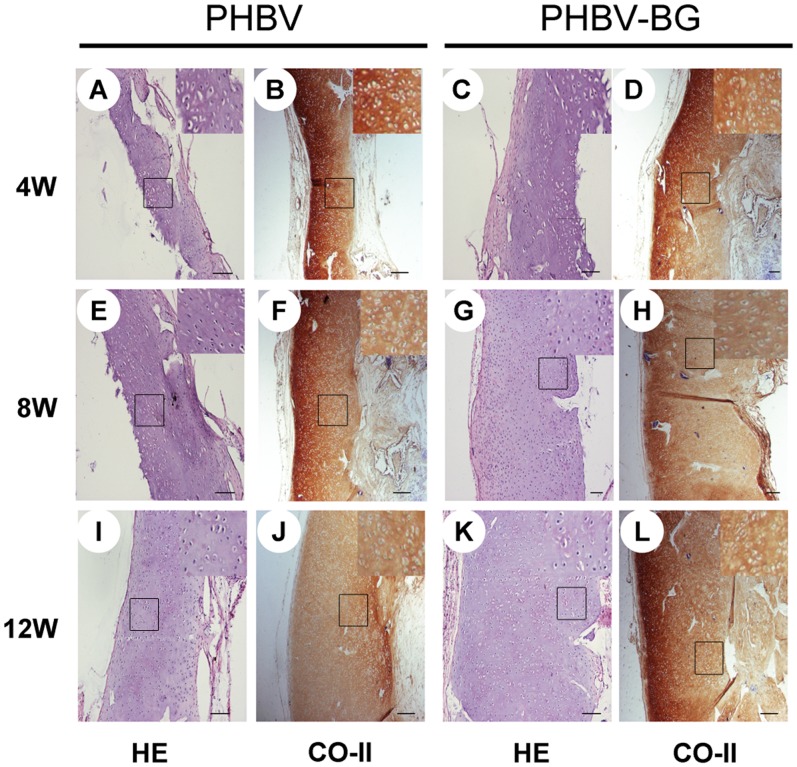
Full-thickness histological images of *in vivo* engineered cartilage. The constructs in the PHBV/BG group were much thicker, with thicker cartilage-like tissue layers, than those in the PHBV group. Scale bar  = 100 µm.

Histological and immunohistochemical analysis of the engineered tissue can be used to assess the formation of neocartilage. It was observed that cartilage-like tissue was formed in both groups, with an obvious lacuna structure and strong expression of type II collagen (Fig. 7). With increasing *in vivo* culture time, the cells underwent obvious migration toward the inner regions of the scaffolds. However, the full-thickness histological and immunohistochemical staining clearly revealed that the cell-scaffold constructs in the PHBV/BG group formed much thicker cartilage-like tissue layers than those in the pure PHBV group at different time points (Fig. 7).

## Discussion

The optimal biodegradable scaffold for cartilage tissue engineering should be biocompatible; able to promote attachment, proliferation, and cellular activity; and have an adequate degradation rate. The chondrocytes on the scaffolds should maintain their chondroblastic phenotype and produce extracellular matrix [28], [29], which should eventually replace the scaffolds. The physical and chemical characteristics of scaffolds determine whether the seeded cells will grow and maintain their morphology and phenotype [30], [31]. These characteristics include the texture of the scaffold surfaces in contact with the cells; the presence of pores; the pore structure, size, and distribution; the presence of chemical groups controlling surface hydrophilicity (positively contributes to the expression of the chondrocyte phenotype); surface free energy; and the ability to form ionic bonds with cells [30]–[32]. PHBV, a biodegradable scaffold used for tissue engineering, has been demonstrated to have the potential to be used as a chondrocyte carrier for cartilage engineering, with appropriate biocompatibility and biodegradability [16].

Some previous studies have reported that the hydrophilicity of materials is an important factor affecting cell adhesion and growth and that improving the surface hydrophilicity of materials will improve the interactions between these materials and cells to elicit controlled cellular adhesion and maintain a stable differentiated phenotype [33], [34]. However, recent studies have shown that engineered cartilage grown on scaffolds made of PHBV, a hydrophobic polyester, had inadequate thickness and poor biomechanical properties due to the poor hydrophilicity of PHBV. Some methods have been reported to improve the hydrophilicity of PHBV, including improving the hydrophilicity of PHBV films by plasma treatment [35], improving the cytocompatibility of PHBV mats using ecoflex [36], and modifying PHBV scaffolds by in situ UV (ultraviolet) polymerization [37]. Wang et al. used oxygen and nitrogen plasma treatment to improve the hydrophilicity of the surfaces of PHBV films. [35]. Li et al. investigated the hydrophilicity of PHBV/wollastonite composite scaffolds and found that the incorporation of hydrophilic inorganic materials into hydrophobic polymers is a feasible approach to improve the hydrophilicity of these composites [18].

In the present study, we incorporated 45S5 BG into PHBV to improve the hydrophilicity of the composites. Previous studies have indicated that PHBV/20% BG has good bioactivity, mechanical properties and biodegradation in addition to a 3D porous structure [16]. Therefore, in this studies, we incorporated 20% BG into PHBV to prepare porous composite scaffolds for *in vitro* and *in vivo* studies. The results show that the incorporation of BG into PHBV resulted in more hydrophilic composite scaffolds, as the water contact angle decreased from 65° to 32° with an increase in the BG content of the composites from 0 to 20 wt%. This change indicated that there was an obvious improvement in the surface hydrophilicity. Due to the improvement in the hydrophilicity, the PHBV/BG composites had improved cell adhesion. In addition, the improved hydrophilicity of the composites promoted cell migration into the inner part of engineered tissues. These findings were supported by histological and immunohistochemical analyses of the *in vivo* engineered cartilage. The cell-scaffold constructs in the PHBV/BG group formed much thicker cartilage-like tissue layers than those in the pure PHBV group at different time points. The results of the fluorescence analysis of cell migration showed that the cells migrated toward the inner region of the scaffolds easily with increasing in vitro culture time in the PHBV/BG group. In the PHBV group, the cells primarily assembled on the surfaces of the scaffolds, even after 12 h culture, suggesting that the cells migrated into the inner part of the engineered tissues after subcutaneous implantation in the PHBV/BG group.

In addition, the incorporation of bioactive inorganic materials into polymers improved the mechanical strength of the scaffold. Li et al. incorporated wollastonite into PHBV to prepare composite scaffolds using the same method, and the results showed that the compressive yield strength of the scaffolds increased from 0.16 to 0.20 MPa with increased wollastonite content from 0 to 20 wt% [18]. In the current study, the compressive yield strength of the PHBV/BG composite scaffolds was significantly improved by the incorporation of the BG particles compared with that of the pure PHBV scaffolds. These results indicate that there was an obvious improvement in the compressive yield strength when the composition of BG in the scaffold increased from 0 to 20 wt%. As both the PHBV and PHBV/BG scaffolds had the same size, porosity and volume, the significant difference in the compressive yield strength between the pure PHBV and PHBV/BG scaffolds was primarily caused by the addition of the BG, which indicated that BG has a strengthening effect on PHBV scaffolds. However, the mechanism should be investigated in a future study. These results were further supported by the comp. modulus of the *in vivo* specimens for the two groups at different time points. The comp. modulus in the PHBV/BG group was significantly higher than that in the PHBV group at different time points. It can be reasoned that the thick cartilage layer formed in the PHBV/BG group contributed to the improved mechanical properties of the neocartilage tissue, which is determined by the content of ECM. It has been reported that the ECM content (GAG content and total collagen content) and the homogeneous structure in the PHBV/BG group contributed to the improvement in the mechanical strength [38]–[40]. Our study also found that the GAG content and the total collagen content in the PHBV/BG group were significantly higher than those in the PHBV group at different time points. Some previous studies have reported that the improved surface hydrophilicity of materials improves the interactions between the composites and cells to facilitate controlled cellular adhesion and maintain a differentiated phenotype [33], [34]. Then, the scaffolds supported the growth of the cells, which maintained their activity, fully expressed their phenotype, produced extracellular matrix, and preserved the characteristic spherical morphology associated with the synthesis of type II collagen and cartilage-specific proteoglycans.

The present study indicated that the incorporation of BG into PHBV could promote cell migration and mass transport and thus resulted in a much thicker layer and improved mechanical strength of the *in vivo* cartilage than pure PHBV. These improvements were due to the improved hydrophilicity of the composites and the characteristics of BG. As shown in the results, most of the chondrocytes in the PHBV/BG group migrated to the inner part of the construct and formed relatively compact cartilage-like tissue with a thicker cartilage layer, which led to a relatively homogeneous structure and increases in the thickness, side length, wet weight and volume.

As a type of inorganic material, bioactive glass is not normally found in bone or cartilage tissue, but it has been used in tissue engineering. Previous studies [41], [42] have shown that bioactive glass is able to promote the growth and proliferation of osteoblasts. Suominen et al. [43] reported that Bioglass led to the direct lamellar bone repair of subchondral bone and the restoration of articular surfaces with primarily hyaline-like cartilage in 12 weeks in rabbit femur osteochondral defects. Sonny et al. [44] reported that bioactive glass was superior to bone allografts with respect to integrating into the adjacent host bone, regenerating hyaline-like tissue at the graft surface, and expressing type II collagen in the articular cartilage. In their study, macroscopic and histological examination did not show any evidence of an immune response to the materials used in this study at 12 weeks in rabbits. In our recent study, chondrocytes were grown on composite scaffolds in vitro and in vivo for weeks to regenerate cartilaginous tissue, and the composite scaffold was also biomimetic and bioactive. We did not observe any adverse effects during cartilage regeneration. The PHBV-Bioglass composite is thus expected to have potential implications as a scaffold for cartilage tissue engineering. Cell metabolism, material degradation, ion deposition and other complex reactions can occur during the repair process, and the further studies are needed to determine the specific mechanisms.

The present results confirm that composite scaffolds of PHBV with bioactive glass have improved hydrophilicity and mechanical properties and produced neocartilage with better biochemical and biomechanical properties. It can therefore be concluded that the PHBV/BG scaffolds prepared in our work are more suitable than pure PHBV for use as chondrocyte carriers in cartilage engineering.

### Conclusions

The addition of bioactive glass to PHBV to form composite scaffolds improved the hydrophilicity of the scaffold surface. The water contact angles suggested that the incorporation of bioactive glass into PHBV could improve the hydrophilicity of the composites, and this enhancement was dependent on the glass content. The composite scaffolds could efficiently promote cell migration toward the inner region of the constructs. With increasing culture time *in vivo*, the chondrocyte scaffold constructs in the composite scaffold group formed thicker cartilage layers with a more homogeneous structure, better mechanical properties, and higher cartilage matrix contents than the constructs in the pure PHBV group. All of these results suggest that the incorporation of bioactive glass is a useful approach to obtain composite scaffolds with improved properties and that the use of these scaffolds can efficiently improve the structure and function of engineered cartilage compared with pure PHBV scaffolds. The incorporation of bioactive glass is thus a useful approach for the development of porous biodegradable scaffolds with improved properties for cartilage tissue engineering.
